# Building character strengths and virtues in Sri Lanka: a cluster randomized pre-post evaluation of a school-based intervention

**DOI:** 10.1186/s12889-025-25752-z

**Published:** 2025-12-23

**Authors:** Suhail Asrar, Miyuru Chandradasa, Sonali Amarasekera, Angela Paric, Sivunadipathige Sumanasiri, Nisha Ravindran, Shehan Williams, Arun Ravindran

**Affiliations:** 1 Department of Psychiatry, University of Toronto, 250 College Street, Toronto, ON M5T 1R8 Canada; 2 Department of Psychiatry, Faculty Of Medicine, University of Kelaniya, Ragama, Sri Lanka; 3 Centre for Addiction and Mental Health, 1000 Queen St W, Toronto, ON M6J 1H4 Canada

**Keywords:** Character development, Youth resilience, School-based program, Web-based intervention, Sri Lanka

## Abstract

**Background:**

Sri Lanka (SL) is a multiethnic nation that has endured a decades-long civil war and a devastating tsunami. These events led to a widespread loss of life, displacement, destruction of family and social infrastructure, and economic collapse. Their impact was compounded by adverse social determinants such as poverty, unemployment, food insecurity, and homelessness. Sri Lankan youth are particularly vulnerable to the cumulative effects of these acute and chronic stressors, underscoring the urgent need for interventions that foster resilience and healing.

**Methods:**

We will implement a 10-week school-based program - Leadership, Empathy, Altruism, Personal Growth, and Social Responsibility (LEAPS) - to cultivate character strengths in youth. The program will be culturally and historically tailored to the SL context, and will integrate key tenets from the country’s four major religions to promote unity, social values, and spiritual collaboration. LEAPS will consist of 10 interactive modules delivered through a web-based platform during regular school hours, supplementing the standard curriculum. Teachers will be trained using the train-the-trainer model to facilitate student engagement, ensure program fidelity and long-term sustainability. The impact of LEAPS on student character development and well-being will be evaluated using a cluster randomized pre-post design. Students will be assigned either to the intervention group (where they will participate in the LEAPS program alongside their regular curriculum) or the control group (where they will follow the standard curriculum without additional interventions). Assessments of character strengths and well-being will be conducted via student questionnaires at baseline, post-intervention, and at a 6-month follow-up. Changes over time and between groups will be analyzed to determine program benefits and effectiveness.

**Discussion:**

The LEAPS program aims to enhance character development and well-being in SL youth. The interactive, web-based format of the program is also anticipated to facilitate uptake, knowledge translation, scalability and long-term sustainability, benefiting not only youth but also their families and broader communities.

**Trial registration:**

This trial was registered with the Sri Lanka Trials Registry (No: SLCTR/2020/016) on June 25, 2020.

**Supplementary Information:**

The online version contains supplementary material available at 10.1186/s12889-025-25752-z.

## Background

Sri Lanka, a lower-middle-income country in South Asia, is home to a diverse population of 20 million people, including Sinhalese, Tamils, Moors, and Burghers [1]. Its history has been shaped by both man-made and natural disasters, notably a decades-long civil war and the devastating Indian Ocean Tsunami in 2004. These events resulted in widespread loss of life and displacement, and significantly disrupted the country’s structural and societal developments [2–4].

Young people, who represent nearly a quarter of Sri Lanka’s population, are especially vulnerable to the negative effects of trauma [5–8]. For example, adolescents who survived the 2004 tsunami reported significant emotional and behavioural issues, as well as symptoms of functional impairment that persisted for up to five years post-disaster [9]. Additionally, social adversities like poverty are exacerbated by the country’s ongoing economic and political instability, and further magnified by the lingering impact of past trauma [9]. These challenges underscore the need for evidence-based, cost-effective interventions that support positive youth development (PYD) in Sri Lanka, where such programs remain notably limited. The proposed study aims to address this gap by implementing and evaluating a school-based initiative designed to enhance character strengths and psychosocial competence among Sri Lankan youth.

### Character development

Interventions based on PYD have been shown to help improve health and functional outcomes in youth. Unlike traditional approaches, PYD focuses on cultivating strengths rather than addressing weaknesses [10, 11], thereby fostering positive emotions, cognitive growth, and behavioural improvements [11]. One PYD model specifically targets the development of one or more of the 24 character strengths identified by Peterson and Seligman [12].

Character strengths not only contribute to positive health outcomes but also act as protective factors against the adverse effects of stressful life events, which can compromise mental, physical, and social well-being [13, 14]. For instance, certain character strengths have been linked to accelerated cardiovascular recovery following acute stressors [15]. Several meta-analyses and systematic reviews have demonstrated the benefits of nurturing character strengths, with many school-based programs demonstrating good efficacy and participant engagement [16–19]. One such example is a 12-week intervention conducted in Northern India, where schools aimed to foster character strengths and well-being among 7th- and 8th-grade students. Following the intervention, students in the experimental group reported greater life satisfaction, happiness, and positive affect compared to the control group [20].

### School environments

Schools have proven to be effective and trusted environments for engaging youth and implementing innovative programs, owing to their feasibility, acceptability, adherence, and sustainability [21–24]. Sri Lanka has a well-established school system, encompassing both secular and religious institutions, which has led to growing interest in integrating initiatives that promote the development of character strengths. This approach is grounded in the belief that strong character is essential for personal growth and serves as the foundation of a moral society [25]. Teachers play a pivotal role in nurturing these traits, as they are often trusted figures from whom youth are willing to seek guidance [26].

Effective youth programs also take into account the broader context of youth development, including family, community, and cultural influences. These programs typically build on young people’s strengths and encourage active participation across all components of the initiative. Research highlights the value of an integrated approach that provides support at the community level, such as leadership training and university-community partnerships [27]. However, developing and implementing character strength interventions within educational systems presents challenges, as cultural, environmental, and religious factors shape perceptions of good character, making it difficult for teachers to navigate these complexities [28]. This underscores the importance of adopting context-specific approaches tailored to the unique experiences and needs of Sri Lankan youth.

### Spirituality and its relation to character development

The world’s four largest religions (i.e. Christianity, Islam, Hinduism and Buddhism) account for approximately 75% of global followers. Notably, these same religions are also the most widely practiced in Sri Lanka, albeit in different proportions, with Buddhism having the largest number of adherents followed by Hinduism, Islam and Christianity. One proposed reason for the widespread following of these major religions is their shared emphasis on character development and ethical conduct. A common thread across their teachings of these faiths is the pursuit of self-improvement and a commitment to the ethical treatment of others.

This study involves the development and evaluation of a novel, school-based, and spiritually oriented character development program for early adolescents in Sri Lanka. It will assess the impact of the intervention in promoting values such as understanding, tolerance, empathy, and respect, among others, within a multireligious and multiethnic society.

## Methods

### Study design and study population

The Leadership, Empathy, Altruism, Personal Growth, & Social Responsibility (LEAPS) program will be a school-based intervention that will be implemented and evaluated using a cluster-randomized pre-post study design in the Gampaha district. Gampaha has been selected for its diversity and feasibility. The district offers a multiethnic student population from diverse socioeconomic backgrounds, enhancing the generalizability of the study. Additionally, the presence of the University of Kelaniya within Gampaha ensures proximity and accessibility for researchers, facilitating implementation. The district’s zonal education office has authorized the project and distribution of school invitations. Schools selected for participation possess the necessary IT infrastructure to host the intervention platform. The zonal education office will also obtain approvals from school principals and teachers, and invite students in grades 7–9 (aged 12–14) to participate, ensuring parental consent is obtained beforehand. To participate in the study, students must be able to use web applications and comprehend Sinhalese.

**Fig. 1:**
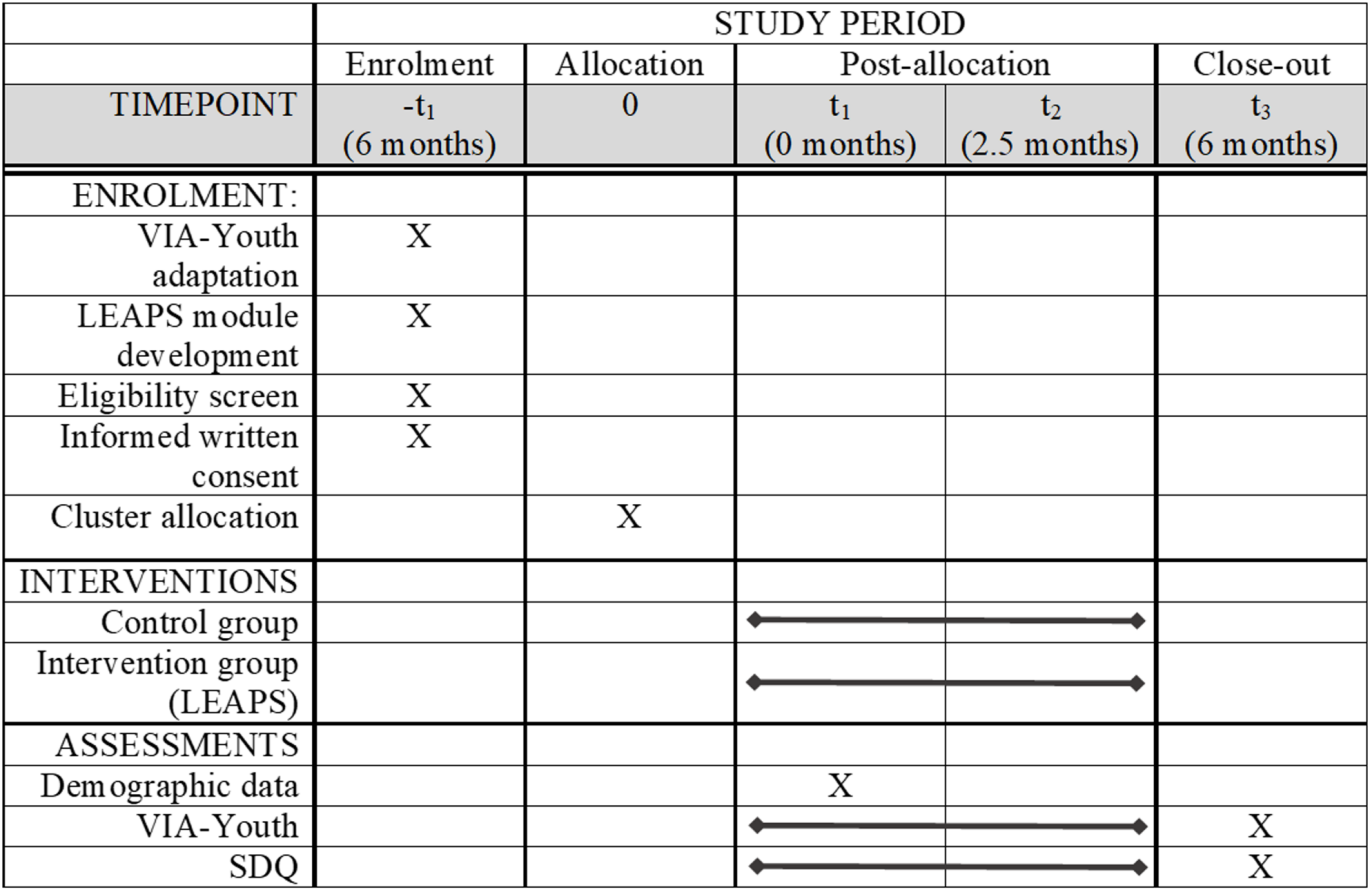
Enrolment, intervention, and assessment schedule for the LEAPS program. Notes. VIA-Youth = Values in Action Inventory for Youth; SDQ = Strengths and Difficulties Questionnaire

After consent is obtained, ten schools will be randomly assigned (using a computer-generated algorithm) to either receive the intervention or be placed on a waitlist. The intervention group will partake in weekly LEAPS sessions during their IT class periods over a 10-week span. The waitlist group will receive the program 3 months after the intervention group begins the study. Both groups will continue receiving their regular school curriculum throughout the study. Assessments will be conducted at three time points: baseline (t_1_), immediately post-intervention (t_2_, 2.5 months), and 6-months post-intervention (t_3_) (see Fig. 1).

### Study aim

The LEAPS program seeks to enhance character development in Sri Lankan youth as a complement to their regular curriculum. It will shift the focus from passive to active learning through student-led, interactive discussions to promote critical thinking. The program emphasizes the shared positive philosophies found in Sri Lanka’s four major religions, using spirituality as a means to foster both social and personal values. The overarching goal of this study is to evaluate the impact of the LEAPS program on character strengths and mental development in youth.

### LEAPS program development

The LEAPS program will consist of interactive sessions that will be designed by a multidisciplinary team of researchers, religious leaders, and clinicians with expertise in youth development in Sri Lanka. A selected software developer will support the program’s technological development and implementation. The unifying moral and spiritual teachings of Sri Lanka’s four major religions will be integrated into the LEAPS curriculum. These teachings will be developed in collaboration with local religious leaders using age-appropriate narratives, images, parables, songs, and other art forms. They will emphasize key character strengths such as tolerance, altruism, respect, kindness, and social responsibility, among others.

The program will be hosted on a user-friendly web platform that provides access to LEAPS materials, interactive games for experiential learning, and online forums for teachers, project teams, and participating students to connect and collaborate. Funding for the development of these materials and digital resources is included in the grant budget. The web application will feature 10 modules focused on character development themes, selected based on the Values-in-Action classification, which outlines 6 core virtues and 24 character strengths (Table 1) [29]. Each module will include 10 interactive segments where students will respond to a statement, paragraph, scenario, image, audio recording or video. Students will receive performance ratings based on their responses and will have the opportunity to retry segments. Upon completing each module, participants will receive a completion award, and at the end of the program, they will be awarded a certificate recognizing their successful completion of the LEAPS character development program.

**Table 1:** Values-in-action classification: 6-core virtues and 24-character strengths

Virtues	Character strengths
Wisdom and Knowledge	Creativity, Curiosity, Judgement, Love of Learning, Perspective
Courage	Bravery, Persistence, Honesty, Zest
Humanity	Love, Kindness, Social intelligence
Justice	Teamwork, Fairness, Leadership
Temperance	Forgiveness, Humility, Prudence, Self-Regulation
Transcendence	Appreciation of beauty and excellence, Gratitude, Hope, Humour, Spirituality

Additionally, a comprehensive training manual will be developed and published by the study team for use by teachers supporting the program. The manual will provide an overview of the importance of character development and character strengths, an outline of the LEAPS modules, and an overview of how the program will be evaluated from student responses. Teachers from participating schools will undergo a 3-day training session (3–4 h per day) using the training manual to enhance their ability to support student engagement, module completion and pre-post survey administration. They will also be encouraged and equipped to train their colleagues in the future. These sessions will be led by Sri Lankan and Canadian teams who will use a “train-the-trainer” approach to support high program fidelity and long-term sustainability. Throughout the intervention, teachers will be encouraged to provide feedback on their experiences and student progress to the research team.

### LEAPS evaluation

To determine program impact, student progress and knowledge uptake will be evaluated through scales that will be administered immediately before and after the intervention, as well as at a 6-month follow-up.

Demographic data will be collected at baseline using an online self-reported questionnaire. This will include participant age, sex, academic performance, ethnicity, religion, language proficiency, region of residence, information related to parents and siblings, and future aspirations. This questionnaire will be developed by the study team.

The Values in Action Inventory for Youth (VIA Youth-96) will be adapted and used as the primary efficacy measure to assess changes in student character strengths. This self-administered questionnaire is designed for individuals aged 10–17 years and uses a 5-point Likert scale with both positively and negatively keyed items [29]. Although the scale has been previously adapted for use in diverse communities and cultures, a Sinhalese version is not yet available. In this study, it will be translated into Sinhalese using forward-back translation consistent with WHO guidelines (WHO, 2016). To ensure conceptual and cultural equivalence, the scale will also be adapted through a structured Delphi consensus process, a widely accepted technique for cross-cultural instrument adaptation [30–32]. The Delphi panel will include 30 experts in child and adolescent mental health, education, and community health, including eight board-certified child and adolescent psychiatrists and consultant general adult psychiatrists, as well as pediatricians, community physicians, school counselors, and psychologists. Through multiple iterative rounds, the panel will refine the translated items to enhance cultural relevance, clarity, and developmental appropriateness for Sri Lankan adolescents. Following adaptation, the Sinhalese VIA-Youth will be validated in our sample by assessing reliability, factor structure, and construct validity.

Changes in behavioural and emotional states will be assessed pre- and post-intervention using the self-report version of the Strengths and Difficulties Questionnaire (SDQ), a brief behavioural screening tool. The SDQ includes 25 items across five subscales: emotional symptoms, conduct problems, hyperactivity/inattention, peer relationship problems, and prosocial behaviour. Although the SDQ has been translated and studied in Sri Lankan samples, the psychometric properties varied [33]. Therefore, we will report its reliability, factor structure, and construct validity in our sample. The questionnaire will be administered at each data collection point.

### Data analysis

Analyses will employ linear mixed-effects models (LMMs) to evaluate changes in VIA-Youth questionnaire and SDQ responses over time and between intervention and control groups, accounting for the nested structure of students within schools. A random intercept for each school will be included to adjust for intra-cluster correlation. Fixed effects will include intervention group, time, student sex, and grade, with interaction terms used to explore whether the intervention effect varies across these factors.

All statistical analyses will be performed using SPSS. The significance level will be set at α = 0.05, and results will be reported with 95% confidence intervals.

### Power and sample size determination

Twenty schools in the Gampaha district of Sri Lanka have the necessary digital infrastructure to support the intervention. The study team will randomly assign ten schools to the intervention arm and ten to the control arm. An a priori power analysis for a two-arm cluster randomized design has been conducted [34–36] to confirm the feasibility of using 20 clusters to detect a small effect size (Cohen’s *d* = 0.25; 37) with a desired power of 90% and a significance level of 0.05.

Under individual randomization, we used G*Power 3.1 (two-sample t test: difference between two independent means, two-tailed) with *d* = 0.25, α = 0.05, and power = 0.90, yielding a required sample size of ~ 337 participants per arm. To adjust for clustering, we assumed an intraclass correlation coefficient (ICC) of 0.01 based on estimates from previous school-based interventions [38]. Assuming an average of 150 students per school agree to participate, the design effect was calculated as:

$$\begin{aligned} \mathrm{Design}\;\mathrm{effect}\;&=\;1\;+\;\lbrack(\mathrm m-1)\;\times\;\mathrm{ICC}\rbrack\;\\&=\;1\;+\;\lbrack(150-1)\;\times\;0.01\rbrack\;\\&=\;2.49 \end{aligned}$$

After multiplying the sample size by this design effect, we obtained the adjusted sample size of 839 per arm. To account for an anticipated 30% attrition rate, the study team will recruit approximately 1,200 students per arm (2,400 total). While simulation-based power analyses are ideal for clustered and repeated data, they require parameter estimates and resources that are currently unavailable to the study team. In this context, applying the design effect to an individual-level calculation is a transparent and widely accepted approach in cluster-randomized trials [34–36].

### Randomization

To assign the 20 schools to the intervention and control arms, allocation will be generated using a computer-based random sequence prepared by an independent statistician. Randomization will be 1:1 and stratified by school size (based on number of eligible students), with allocation concealed until assignment.

### Ethical considerations

The study has been approved by the ethics board at the University of Kelaniya and registered with the Sri Lanka Trials Registry (No: *SLCTR/2020/016*). The authors obtained permission from the Western Province Department of Education, relevant Zonal Education Offices for the District of Gampaha, and from principals of the selected schools to recruit teachers and students. To obtain parental consent, researchers and teachers will provide students with (1) an information sheet, (2) a parental consent form, and (3) an opt-out form for parents who do not wish for their child to participate. All students will be informed that participation is voluntary, that LEAPS is not part of their regular syllabus, and that they can withdraw their consent and leave the study at any time.

Participants will be assigned a unique, anonymous code that will be used on all their data forms. Electronic data will be stored on password-protected computers and hard copies will be stored in locked storage facilities at the University of Kelaniya. Access to these computers and facilities will be restricted to members of the research team. Consent forms will be stored separately to prevent linking participant names with their codes and associated data.

## Discussion

Research over the past two decades suggests that PYD approaches can positively influence mental well-being, to which character development likely contributes. In post-war Sri Lankan society, character-strengthening initiatives may contribute to youth development, offering potential benefits at individual, familial and societal levels [39]. PYD-focused programs have been associated with improvements in academic performance, employment outcomes, and other functional domains, and appear to foster resilience and moral development [37, 39, 40]. Importantly, cultural adaptation is well documented to enhance the uptake and effectiveness of societal interventions [41]. As such, the development of LEAPS will incorporate linguistic, ethnic, and religious adaptations during program design and implementation, with ongoing opportunities for stakeholder input. We anticipate that participation in LEAPS could be associated with increased tolerance, altruism, kindness, and social intelligence among Sri Lankan youth. We will also explore the potential for scaling the program to other provinces of Sri Lanka in collaboration with the Ministries of Health and Education.

### Dissemination

The LEAPS program will be made freely available on the University of Kelaniya website. Results will be shared in aggregate format (e.g. infographics) with participating students, teachers, and community members, and presented to key stakeholders through virtual or in-person meetings. Findings will also be disseminated through publications and presentations at several national and international conferences.

## Supplementary Information

Supplementary Material 1.

## Data Availability

No datasets were generated or analysed during the current study.
